# Freshwater Bacterioplankton Metacommunity Structure Along Urbanization Gradients in Belgium

**DOI:** 10.3389/fmicb.2019.00743

**Published:** 2019-04-12

**Authors:** Fabio Toshiro T. Hanashiro, Shinjini Mukherjee, Caroline Souffreau, Jessie Engelen, Kristien I. Brans, Pieter Busschaert, Luc De Meester

**Affiliations:** ^1^Laboratory of Aquatic Ecology, Evolution and Conservation, Department of Biology, KU Leuven, Leuven, Belgium; ^2^Department of Gynaecology and Obstetrics, UZ Leuven, Leuven, Belgium; ^3^Division of Gynaecological Oncology, Leuven Cancer Institute, KU Leuven, Leuven, Belgium

**Keywords:** urbanization, freshwater bacterioplankton, metacommunity, spatial scales, shallow ponds

## Abstract

Urbanization is transforming and fragmenting natural environments worldwide, driving changes in biological communities through alterations in local environmental conditions as well as by changing the capacity of species to reach specific habitats. While the majority of earlier studies have been performed on higher plants and animals, it is crucial to increase our insight on microbial responses to urbanization across different spatial scales. Here, using a metacommunity approach, we evaluated the effects of urbanization on bacterioplankton communities in 50 shallow ponds in Belgium (Flanders region), one of the most urbanized areas in Northwest Europe. We estimated the relative importance of local environmental factors (35 abiotic and biotic variables), regional spatial factors and urbanization (built-up area) quantified at two spatial scales (200 m × 200 m and 3 km × 3 km). We show that urbanization at local or regional scales did not lead to strong changes in community composition and taxon diversity of bacterioplankton. Urbanization at regional scale (3 km × 3 km) explained only 2% of community composition variation while at local scale (200 m × 200 m), no effect was detected. Local environmental factors explained 13% (OTUs with relative abundance ≥ 0.1%) to 24% (12 dominant OTUs -≥ 1%) of community variation. Six local environmental variables significantly explained variation in bacterioplankton community composition: pH, alkalinity, conductivity, total phosphorus, abundance of *Daphnia* and concentration of copper (Cu), of which pH was partly mediated by urbanization. Our results indicate that environmental rather than spatial factors accounted for the variation in bacterioplankton community structure, suggesting that species sorting is the main process explaining bacterioplankton community assembly. Apparently, urbanization does not have a direct and strong effect on bacterioplankton metacommunity structure, probably due to the capacity of these organisms to adapt toward and colonize habitats with different environmental conditions and due to their fast adaptation and metabolic versatility. Thus, bacterioplankton communities inhabiting shallow ponds may be less affected by environmental conditions resulting from urbanization as compared to the impacts previously described for other taxa.

## Introduction

The extent of anthropogenic transformation of biotic and abiotic conditions on Earth is so profound that we have now entered a new geological epoch, aptly dubbed the Anthropocene (Lewis and Maslin, 2015; Waters et al., 2016). With the dramatic expansion of urban land area and the global urban human population over the last few decades, urbanization has become one of the most extreme forms of land-use alterations triggered by humans (Grimm et al., 2008). Urban areas fundamentally differ from natural areas in terms of atmospheric chemistry, geochemistry, climate, hydrology and vegetation cover and thus form unique ecosystems (Alberti et al., 2003; Kaye et al., 2006). Consequently, there is a growing need to better understand the impact of urbanization on biodiversity and ecosystem functioning.

The relationship between urbanization and biodiversity is multifaceted. Urbanization can affect species richness, evenness and composition by means of several direct and indirect mechanisms. Habitat loss and fragmentation caused by urbanization might lead to reduced species richness and evenness thereby causing biotic homogenization (McKinney, 2006). An increase in impervious surface may also result in reduced species richness and changes in species composition through physical and chemical changes due to alterations of the hydrologic regime and polluted run-off (Morse et al., 2003; Chadwick et al., 2006). The introduction of exotic species by humans in cities (e.g., for cultivation purposes and by accidental importation) may cause replacement of native species (McKinney, 2008). In addition to changes in species composition, a recent meta-analysis has shown that urbanization increases the rates of phenotypic change across different taxa (Alberti et al., 2017).

However, depending on the taxonomic group, the level of urbanization and the spatial scale investigated, contrasting patterns in responses of community composition and trait change to urbanization have been reported (McKinney, 2008). The majority of earlier studies have been performed on higher plants and animals, with only a limited number of studies so far focusing on microbial responses to urbanization in soil (Xu et al., 2014; Reese et al., 2015), artificial surfaces (Afshinnekoo et al., 2015), streams (Teittinen et al., 2015) and lakes (Fisher et al., 2015; Newton and McLellan, 2015). Considering the tremendous diversity of bacteria (Hug et al., 2016) and their key role in major ecosystem services (Falkowski et al., 2008; Van Der Heijden et al., 2008), it is crucial to increase our insight on microbial responses to urbanization. So far, studies on microbial diversity in the context of urbanization have primarily focused on describing patterns of species richness and composition in specific cities and towns (Xu et al., 2014; Afshinnekoo et al., 2015; Reese et al., 2015). In order to develop a cohesive theory on how microbes respond to urbanization, a better understanding of ecological processes underlying the observed responses is needed.

High dispersal rates, short generation times and rapid adaptation to environmental stressors are some of the important traits of microbes that set them apart from the majority of macro-organisms (Andersson et al., 2014). Species responses to urbanization have been shown to depend on dispersal rates and degree of habitat specialization that might in turn influence metacommunity dynamics, with implications at multiple spatial scales (Concepción et al., 2015) Urban ecological footprints can be observed at scales ranging from local (altered abiotic and biotic conditions) to regional (altered habitat heterogeneity and connectivity) and therefore biodiversity responses to urbanization should best be considered within a hierarchical metacommunity framework (Johnson et al., 2013). The metacommunity framework summarizes the relative contribution of local and regional processes in regulating the composition of ecological communities (Leibold et al., 2004). Most studies have reported species sorting as the predominant mechanism shaping microbial communities (Van der Gucht et al., 2007; Lindström and Langenheder, 2012; Souffreau et al., 2015; Muscarella et al., 2016). Species sorting emphasizes the role of local abiotic and biotic conditions in determining community composition and requires dispersal rates that are sufficiently high to allow for species to reach the habitats with their preferred niches but not so high to cause homogenization via mass effects (Leibold et al., 2004). Species sorting likely predominates in bacterial communities because of a combination of high dispersal rates and fast local population growth rates (Van der Gucht et al., 2007). Some studies, however, have provided evidence for neutral dynamics, highlighting the role of regional factors in regulating community assembly in microbes (Drakare and Liess, 2010; Langenheder and Székely, 2011; Langenheder et al., 2012). Others observed spatially limited distribution patterns in microbial species, ranging from centimeters to 100s of kilometers (Martiny et al., 2006) and being the result of historical processes (e.g. drift, priority effects, and past environmental conditions) (Fukami et al., 2010; Andersson et al., 2014). In addition, a synergistic interaction of species sorting and neutral processes has also been detected (Langenheder and Székely, 2011; Liu et al., 2015).

Wetlands, ponds and lakes provide important ecosystem services to society and these services are to a large extent mediated by microbial communities (e.g., water purification, degradation of xenobiotics, and nutrient recycling) (Kremen, 2005). Ponds are ideal model systems for the study of ecology, evolution, and conservation in a metacommunity context because they often show contrasting ecological conditions and have clearly delimited boundaries in the landscape (De Meester et al., 2005).

In this study, we investigated the structure and composition of bacterioplankton communities in freshwater ponds located along well-characterized urbanization gradients in Belgium in order to get insight into the ecological processes acting on bacterioplankton in an urbanization context. We employed a spatially hierarchical sampling site selection to capture the combined as well as independent influence of environmental and spatial processes on bacterioplankton metacommunity structure across strong gradients of urbanization at two different spatial scales. Using freshwater ponds as study systems, we tested the follow hypotheses: (1) urbanization would lead to more homogeneous bacterioplankton community composition, with relatively minor effects on diversity; (2) in urban landscapes, environmental factors would be relatively stronger than spatial factors in regulating bacterioplankton community assembly. This implies that fragmentation effects captured by an increase in urbanization along the gradient (measured using the hierarchical design) are less important compared to changes in local conditions (abiotic and biotic factors) mediated by urbanization.

## Materials and Methods

### Hierarchical Sampling Design and Bacterioplankton Sampling

Sampling was carried out within a polygon broadly defined by the cities of Ghent, Antwerp, Leuven, and Brussels (approximately 5000 km^2^; Belgium) during summer 2013. A GIS-based (built-up area, i.e., percentage of area covered by buildings – data from *Grootschalig Referentie Bestand Vlaanderen* 2013; scale: 1/250-1/5000, Agency of Geographical Information Flanders [AGIV]) site selection was performed to select 27 plots of 3 km × 3 km, categorized based on the percentage of built-up area as high (>15%; 9 plots), medium (5–10%; 9 plots) and low (<3%; 9 plots) urbanized plots (a detailed map of the sampling design is available in Supplementary Figure S1). Within each of these 27 plots, three subplots were selected representing the same three levels of urbanization (high, medium, and low) at the scale of 200 m × 200 m, resulting in 81 subplots. In this way, it was possible to capture the influence of three levels of urbanization (low, medium, and high) at two different spatial scales. As built-up area is the area occupied by buildings, excluding streets, parking lots, etc., a >15% built-up area represents a high level of urbanization. In each of the 81 subplots, a shallow pond (area < 1 ha, depth ≤ 3 m, fishless) was selected for sampling. After leaving out samples with insufficient coverage of sequences, 50 shallow ponds from the original design (81 shallow ponds) were analyzed in this study. The resulting dataset is to some degree unbalanced toward the different urbanization categories, but the imbalance is moderate (see Supplementary Table S1).

The field survey was performed between end of May and beginning of July 2013, and each pond was sampled once. To sample water for characterization of bacterioplankton (defined as bacterial communities that drift along the water column) and abiotic variables, we collected a depth integrated water sample (in total 15 L) with a pre-rinsed tube sampler (length: 1.85 m; diameter: 75 mm) in the central area of each pond. We pre-filtered pond water using a 2 mm mesh size sieve to remove larger materials. Samples were kept at 4°C in 2 L dark jars until they were further processed at the laboratory on the same day of sampling. For bacterioplankton characterization, we filtered up to 100 mL of pond water through a MCE filter with 0.22 μm pore size (Milipore^®^, United States) and the samples were stored at -80°C until DNA extraction.

### Measurement of Abiotic and Biotic Variables

To characterize local environmental biotic and abiotic factors, we utilized standardized protocols following Declerck et al. (2006) and De Bie et al. (2012), summarized as follows. In the field, pH, conductivity, water temperature and dissolved oxygen were measured using a Hach^®^ multimeter (United States). For measuring transparency, we used a Snell tube, an adaptation of the classic Secchi disk method. In brief, we added water to a dark gray plastic tube (length: 60 cm; diameter: 8 cm) and assessed the depth at which a small 5 cm cross-section Secchi disk could be seen in that tube. This allows to measure transparency even in shallow systems where the Secchi depth would exceed water depth. Shaded pond area was visually estimated, and sludge depth, maximum depth and bank angle were measured using a graduated scale. Surface area of the ponds was estimated in Google Earth (version 7.1.8, United States). A table with summarized pond morphometric data is available in Supplementary Table S2.

We measured total phosphorus concentration of the water samples with the ascorbic acid method after perchlorate digestion (Murphy and Riley, 1962) and total nitrogen concentration using the Kjeldahl method (United States Environmental Protection Agency [USEPA], 1983). The concentration of available phosphorus and nitrogen (dissolved nutrients) was determined after filtration of 50 mL of water on a GF/F filter with a Technicon autoanalyser III (United Kingdom). Dissolved metals (Cu, Ni, Zn, Al, Cr, Mg, Fe, Mo, As, Ag, Cd, and Pb) were quantified using a ICP-MS (Inductively Coupled Plasma Mass Spectrometry) (Agilent 7700x ICP-MS) (United States) after filtration over a 0.22 μm pore size sterile filter. Alkalinity and the concentration of sulfates were measured in the laboratory following standardized protocols using Hach^®^ Water Analysis (Hach, 1992) (United States). Dissolved organic carbon was estimated as the NPOC (Non-purgeable Organic Carbon) by catalytic reaction and infrared detection of the carbon dioxide formed after filtration of 15 mL of water on a 0.45 μm pore size filter. For the measurement of suspended matter, we filtered water samples (up to 1 L) over a GF/F filter that was previously dried at 105°C for 6 h and weighted. After filtration of the water samples, the filters were dried again following the same procedure and the difference of weight between the pre-weighted and the dried filter was recorded as suspended mater content.

We also incorporated biotic variables in our analyses: concentration of chlorophyll-a (estimated with a calibrated Aquafluor^®^ Turner Designs fluorometer – United States), zooplankton abundance, *Daphnia* abundance, biomass of *D. magna*, and *Ceriodaphnia* abundance. We sampled zooplankton using a tube sampler (length: 1.85 m; diameter: 75 mm), collecting in total 60 L of depth-integrated water sample at the pelagic and littoral zones from each pond. We filtered 20 to 40 L of this volume over a 64 μm mesh size sieve. Samples were fixed with formalin (7%) and stored in a 60 ml vials for species identification in the laboratory. Zooplankton identification was conducted using a stereomicroscope and a minimum of 300 individuals were identified per sample (Gianuca et al., 2018). Zooplankton abundances were calculated as number of individuals per liter sample. Zooplankton, bacterioplankton and environmental sampling were performed on the same day for a specific pond.

### DNA Extraction, PCR of 16S rRNA Genes and Sequencing

DNA was extracted using the UltraClean^TM^ Soil DNA isolation kit (MoBio Laboratories, Inc., Carlsbad, CA, United States) and amplified by PCR using the 16S rRNA gene universal eubacterial primers E338F (5′-ACTCCTACGGGAGGCAGCAGT-3′) and E797R (5′-GGGTATCTAATCCTG-3′) (amplicon length of 459-bp covering the variable V3 region of the 16S rRNA gene). The PCR mixtures were prepared with 2.5 μl 10x PCR buffer (Eurogentec, Seraing, Belgium), 1 μl MgCl_2_ (50 mM; Eurogentec), 2.5 μl dNTPs (2 mM; Thermo Scientific, Waltham, MA, United States), 1.0 μl of each primer (20 pmol μl^-1^ each; Eurogentec), 0.2 μl Silverstar *Taq* DNA polymerase (5.0 U μl^-1^; Eurogentec) and 1 μl standardized template DNA (10.74 ng μl^-1^) in a total reaction volume of 25 μL. The PCR was performed with the follow temperature/time conditions: initial denaturation at 94°C for 2 min, 30 cycles of 94°C for 1 min, 55°C for 1 min and 72°C for 1 min, and a final extension for 5 min (72°C). PCR products were evaluated on gel electrophoresis, the bands then were excised and purification was performed using a Qiaquick Gel extraction kit (Qiagen, Venlo, Netherlands). For the quantification of the PCR products, we used the PicoGreen dsDNA Assay Kit (Life Technologies, United States). Sequencing was performed at the Genomics Core of KU Leuven (Belgium) using a Roche 454 GS FLX+ (Switzerland).

### Processing 454 Pyrosequecing Data

We processed the 16S rRNA gene pyrosequences using the software mothur (version 1.30.2, United States). The sequences contained in the flowgrams (SFF file) were sorted into groups according to the barcode sequences. The standard operating procedure^1^ was followed for the analysis (Schloss et al., 2011). A total of 89015 good-quality sequences were recovered from the 50 samples that were used for downstream analysis. A 97% identity in rRNA gene sequence was used as the operational taxonomic unit (OTU) definition. A total of 1133 OTUs (no global singletons) were identified from the total set of samples. Samples were rarefied to 688 sequences per sample to compare relative differences between samples. Raw sequences from all samples were submitted to the European Nucleotide Archive under the Accession Nos. ERR1871981-ERR1872018.

### Statistical Analysis

We conducted all analyses in R (version 3.2.2, 2014) using the package vegan (Oksanen, 2015). Except for richness, all analyses were performed on relative abundance data. Diversity and exclusiveness analyses were performed using all 1133 OTUs (no global singletons). Since the sequencing depth was relatively low in our study, we assume that we mainly captured the most abundant taxa in our samples. We therefore analyzed these data making two subsets, with different cutoff levels:: ≥ 0.1% (abundant taxa – 100 OTUs) and ≥ 1% (dominant taxa – 12 OTUs) of the rarified total dataset. Additionally, this could be justified because microbial communities are usually composed of a small group of abundant organisms and a vast number of rare taxa (Pedrós-Alió,, 2012; Baltar et al., 2015; Liu et al., 2015). To estimate the impact of urbanization, all the below-mentioned analyses were performed for the two datasets. In order to visualize compositional changes among the different urbanization categories at plot and subplot levels, we used a non-metric multidimensional scaling (NMDS) approach with Bray-Curtis index as measure of dissimilarity. To verify the changes in composition due to urbanization and associated environmental variables, we performed PERMANOVA (Anderson, 2001) using the *adonis* function (number of permutations = 9999, stratum = plot identifier – this parameter was included to account for the spatial dependency of subplots within the same plot) of the vegan package. To evaluate whether variability in composition within samples changed among urbanization categories, we performed PERMDISP (Anderson, 2006) using the function *betadisper* followed by a permutation test of the *F*-values using the function *permutest*. PERMANOVA and permutest analyses were performed using 9999 permutations. The effect of urbanization level at both the local (subplot) and regional (plot) scale and their interaction on the (i) diversity, (ii) abundances of each of the 10 most abundant classes, (iii) abundance of each of the 12 dominant OTUs, and (iv) six selected environmental variables was tested univariately by means of generalized linear mixed models (GLMMs) performed with the function *lme*. Levels of urbanization (low, medium, high) at the subplot and plot scale and their interaction were set as fixed factors. To account for the spatial dependency of subplots within the same plot, a plot identifier was incorporated as a random factor, nested within plot. The tested environmental variables were selected by forward selection for the variation partitioning analysis described below.

The relative contribution of spatial (S) and environmental (E) factors, urbanization level at plot level (U_plot_) and urbanization at subplot level (U_subplot_) in determining local species composition was determined using a variation partitioning approach (Peres-Neto et al., 2006). To identify which variables should be incorporated in the variation partitioning for the environmental and spatial models, we used forward selection (Blanchet et al., 2008). Only significant variables selected by the forward selection (permutation test = 9999, *P* < 0.05) were included in the environmental (E) and the spatial models (S) for the variation partitioning analysis. For the variation partitioning and the forward selection analyses, we used the function *varpart* and *forward.sel*. To assess the pure components [i.e., corrected for the other components, e.g., E|(S + U) is the pure environmental component without spatial and urbanization effects], a permutation test (1000 permutations) was performed using the functions *rda* and *anova.cca*. We also used the *by = terms* command to test which specific variables contributed to the pure environmental model. All environmental variables were first tested for possible collinearity based on Pearson correlation values. When the correlation between two variables was higher than 0.7, only one of the variables was included in the downstream analyses. The selection of variables was also based on existing knowledge on the importance of the different variables in an urbanization context. All environmental variables were log-transformed (except pH) and standardized (z-scores). The spatial component was generated based on the geographical coordinates of each site. To incorporate the non-linear spatial components, we used Moran’s eigenvector map (MEM) (Dray et al., 2006). Latitude and longitude were included as variables to test separately for the linear spatial component. Urbanization levels (three categories) were given as dummy variables. For the community data, we used Hellinger transformation to reduce the impact of zero values in our dataset (Legendre and Gallagher, 2001).

## Results

### Urbanization-Driven Community Composition and Diversity

Despite the low number of sequences per sample (688 sequences), almost the totality of samples reached saturation for the abundant OTUs (relative abundance ≥ 0.1% of the total dataset) (Supplementary Figure S2). This indicates that the abundant fraction of the communities could be accurately characterized using this number of sequences. Besides, performing all the analyses with a coverage of 1000 sequences per sample (38 samples) gave similar results (Supplementary Tables S3–S5).

**FIGURE 1:**
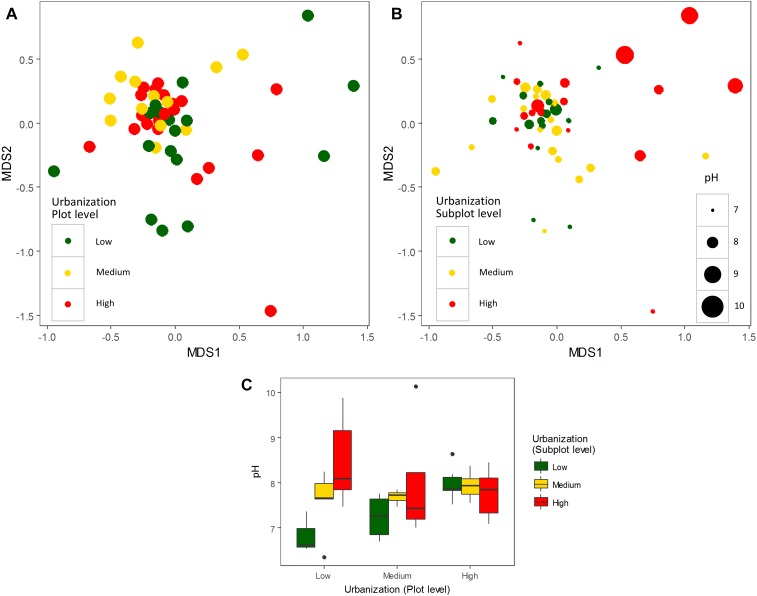
Non-metric multidimensional scaling (NMDS) plot of bacterioplankton communities (abundant OTUs – relative abundance ≥ 0.1%) colored by urbanization categories (low, medium, and high) at plot level **(A)** and subplot level **(B)**. Urbanization categories are represented by colors and pH values represented by the size of each dot for the subplot level. **(C)** Box-plots of pH values across urbanization categories (low, medium, and high) at subplot and plot levels.

An effect of urbanization category (low, medium, and high) on bacterioplankton community composition was detected at plot level by PERMANOVA (*R*^2^ = 0.06, *P* = 0.01) (visualized by NMDS in Figure 1A), whereas at subplot level, no effect of urbanization was detected (*R*^2^ = 0.04, *P* = 0.27) (visualized by NMDS in Figure 1B). No interaction between subplot and plot levels of urbanization on bacterioplankton community composition was detected (*R*^2^ = 0.84, *P* = 0.30) (Supplementary Table S6).

**FIGURE 2:**
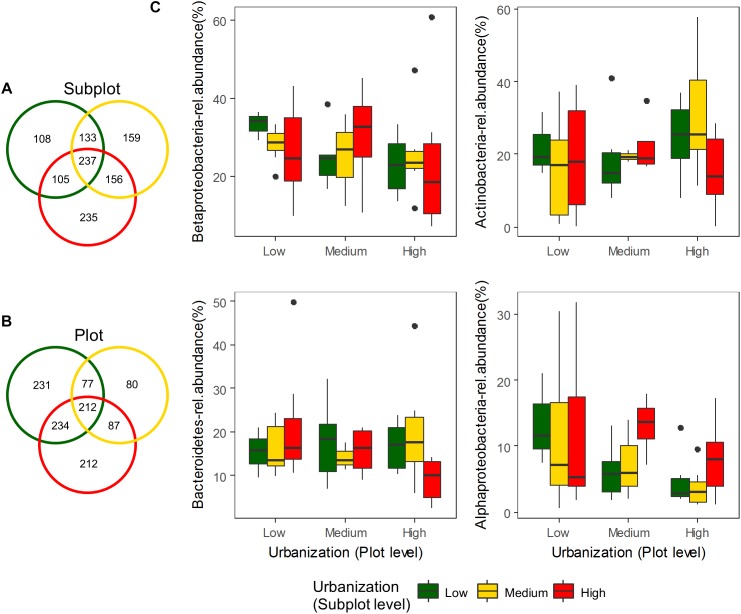
Venn diagrams showing exclusive and shared OTUs for the three urbanization categories (total dataset – 1133 OTUs) at **(A)** subplot level and **(B)** plot level. Box-plots of the four most abundant classes of bacteria found along the three urbanization categories (low, medium, and high) at subplot and plot levels **(C)**.

We did not detect any significant effect of urbanization at subplot or plot levels on bacterioplankton diversity (number of OTUs, Shannon diversity, Chao1 and ACE estimators; Supplementary Figure S3 and Supplementary Table S7). To estimate whether more subtle changes could be detected at more specific levels, we tested the effect of urbanization on relative abundances of the most dominant OTUs, and also separately on phylum and class levels. A total of 17 phyla were detected in the studied ponds for the abundant fraction (relative abundance ≥ 0.1% of the total dataset). Proteobacteria (44.44% of total abundance), Actinobacteria (21.32%), Bacteroidetes (16.63%), and Firmicutes (1.95%) were the most abundant phyla. No significant differences in the relative abundances across urbanization categories were detected for the 10 most abundant classes found in our study at both scales studied (local and regional) (Figure 2C – four most abundant classes depicted) (Supplementary Figure S4 and Supplementary Table S8). Among the OTUs restricted to highly urbanized subplots that were identified to genus level, *Algoriphagus*, *Pedobacter*, and *Flavobacterium* were the most abundant (Table 1). Among the 12 dominant OTUs (>1% of relative abundance), which accounted for 57.92% of the total community composition, two OTUs showed significant differences in relative abundance across urbanization categories (Supplementary Figure S5). An OTU from the class Actinobacteria was more abundant in high urbanization subplots than in medium and low urbanizations subplots and was more abundant in medium urbanization plots. An OTU from the genus *Methylobacter* (Supplementary Table S9) was less abundant in medium than in low and high urbanization subplots (Supplementary Figure S5).

**Table 1:** Bacterioplankton taxa that are restricted (>90% of occurrence) to ponds in highly urbanized subplots (# reads = total number of reads in all ponds).

Phylum	Class	Order	Family	Genus	# reads	EXC (%)
Bacteroidetes	Sphingobacteria	Sphingobacteriales	Cyclobacteriaceae	*Algoriphagus*	38	100
Bacteroidetes	Sphingobacteria	Sphingobacteriales	Sphingobacteriaceae	*Pedobacter*	35	100
Proteobacteria	Epsilonproteobacteria	Campylobacterales	Helicobacteraceae	*Sulfurimonas*	28	100
Bacteroidetes	Flavobacteria	Flavobacteriales	Flavobacteriaceae	*Flavobacterium*	14	100
Actinobacteria	Actinobacteria	Actinomycetales	Unclassified	Unclassified	14	100
Bacteroidetes	Unclassified	Unclassified	Unclassified	Unclassified	13	100
Bacteroidetes	Flavobacteria	Flavobacteriales	Flavobacteriaceae	Unclassified	12	100
Bacteroidetes	Sphingobacteria	Sphingobacteriales	Chitinophagaceae	Unclassified	11	100
Proteobacteria	Alphaproteobacteria	Sphingomonadales	Sphingomonadaceae	*Sandarakinorhabdus*	10	100
Bacteroidetes	Unclassified	Unclassified	Unclassified	Unclassified	10	100
Bacteroidetes	Sphingobacteria	Sphingobacteriales	Chitinophagaceae	Unclassified	10	100
Proteobacteria	Betaproteobacteria	Burkholderiales	Burkholderiaceae	*Limnobacter*	98	98.9
Proteobacteria	Unclassified	Unclassified	Unclassified	Unclassified	56	98.2
Proteobacteria	Epsilonproteobacteria	Campylobacterales	Helicobacteraceae	*Sulfurimonas*	82	96.3
unclassified	Unclassified	Unclassified	Unclassified	Unclassified	72	95.8
Fusobacteria	Fusobacteria	Fusobacteriales	Fusobacteriaceae	*Cetobacterium*	21	95.2
Proteobacteria	Alphaproteobacteria	Rhodospirillales	Acetobacteraceae	*Roseomonas*	39	94.9
Bacteroidetes	Sphingobacteria	Sphingobacteriales	Chitinophagaceae	*Sediminibacterium*	45	93.3
Proteobacteria	Alphaproteobacteria	Rhizobiales	Unclassified	Unclassified	39	92.3
Bacteroidetes	Flavobacteria	Flavobacteriales	Unclassified	Unclassified	13	92.3
Firmicutes	Bacilli	Bacillales	Bacillaceae_1	*Bacillus*	13	92.3
Bacteroidetes	Unclassified	Unclassified	Unclassified	Unclassified	21	90.5


*Taxa are ranked by percentage of exclusiveness (EXC) (ex: high – 100% means only occurrence in ponds situated in highly urbanized subplots).*

In order to estimate other effects of urbanization on bacterioplankton structure, we estimated the number of exclusive OTUs along the gradient and pointed out the most common OTUs at highly urbanized subplots. At the subplot (local 200 m × 200 m) level, there were a higher number of exclusive OTUs in high urbanization ponds (235) compared to low (108) and medium (159) urbanization ponds (Figure 2A). At the plot (regional 3 km × 3 km) level, there were a higher number of exclusive OTUs in low urbanization ponds (231) compared to high (212) and medium (80) urbanization ponds (Figure 2B). For the OTUs exclusive to ponds in highly urbanized subplots, the phylum Bacteroidetes was represented by the families Cyclobacteriaceae, Sphingobacteriaceae, Flavobacteriaceae, Chitinophagaceae, and Sphingomonadaceae (Table 1).

### Relative Impact of Urbanization at Local and Regional Scale on Community Composition

**FIGURE 3:**
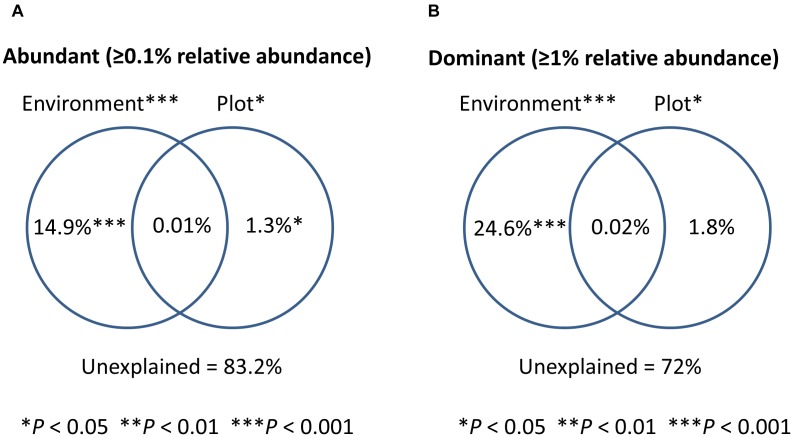
Venn diagrams showing the results of variation partitioning analyses. Percentage of variance explained by different models for the abundant fraction (100 OTUs) is shown in **(A)** and for the dominant OTUs of bacterioplankton community (12 OTUs) is shown in **(B)**.

To test the relative contribution (adjusted *R*^2^ values) of urbanization, environmental and spatial factors in shaping bacterioplankton communities, we performed a variation partitioning analysis. Environmental factors (variables selected by forward selection) and urbanization at plot level explained together 16.8% of the variation in bacterioplankton community composition of the abundant fraction (relative abundance ≥ 0.1%) (Figure 3A). In this model, pure environmental factors explained the highest amount of variation (14.9%, *P* < 0.001), while pure urbanization at plot level explained a small but significant part of the variation (1.3%, *P* < 0.01) (Table 2). For the 12 dominant OTUs (>1% relative abundance), environmental variables and urbanization at plot level explained together 28% of the variation (Figure 3B). Pure environment was the component explaining the largest amount of community variation for the dataset on dominant OTUs (24%, *P* < 0.001) (Table 2). Pure urbanization at plot level (1.8%) was not significant and 1.6% of the variation was shared between environmental factors and urbanization at plot level.

**Table 2:** Summary of the RDA analyses showing the relative importance of the different components of explained variation of community composition in the abundant (100 OTUs – relative abundance ≥ 0.1%) and in the dominant fraction (12 OTUs – relative abundance ≥ 1%).

Model	Components studied							
	Marginal effect				Conditional effect			
	E	S	U(subplot)	U(plot)	E	S	U(subplot)	U(plot)
Abundant (100 OTUs)	0.154^∗∗∗^	NS	NS	0.019^∗^	0.149^∗∗∗^	NS	NS	0.013^∗^
Dominant (12 OTUs)	0.261^∗∗∗^	NS	NS	0.034^∗^	0.246^∗∗∗^	NS	NS	0.018^NS^


*The effects of each component (E, environment; S, space; U, urbanization at subplot and plot levels) are not (marginal effect) or are corrected (conditional effect) for the other components. NS means that a certain model did not explain the variation in community composition significantly, or that the % explained variance was not significant. E, environment; S, space; U, urbanization (NS, not significant, ^∗∗∗^*P* < 0.001, ^∗^*P* < 0.05).*

### Environmental Factors Correlated With Community Structure

**FIGURE 4:**
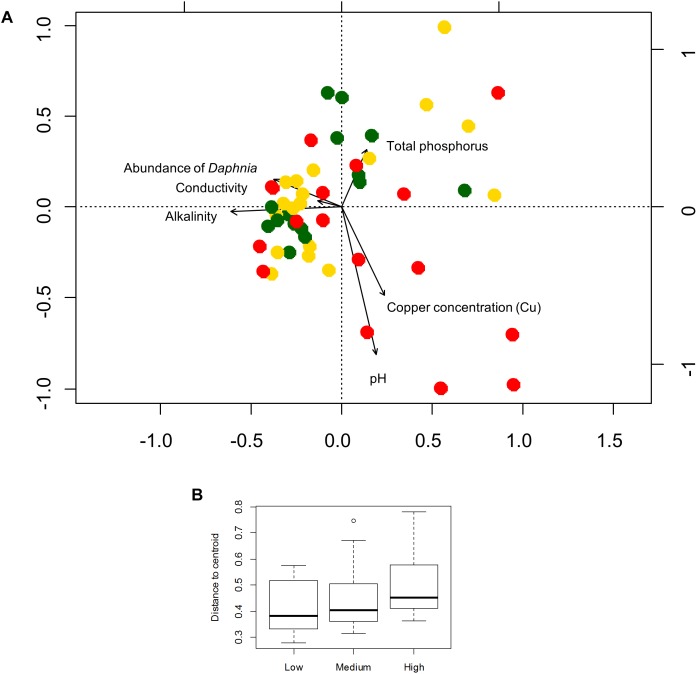
**(A)** Redundancy analysis of the bacterioplankton community composition for the abundant fraction (100 OTUs) and environmental variables selected by forward selection. Colors represent levels of urbanization around the ponds (low, medium, and high level) at subplot level. **(B)** Box-plot showing the variation (in terms of beta diversity) within bacterioplankton communities among the urbanization categories at subplot level. The average distance of a sample to the centroid of each urbanization category was used to calculate an *F*-statistic followed by a PERMUTEST.

Six local environmental variables were selected by forward selection to significantly explain variation in bacterioplankton community composition: conductivity, pH, alkalinity, total phosphorus, abundance of *Daphnia* and concentration of copper (Cu) (Table 3). Based on visual inspection of Figure 4A, bacterioplankton communities in ponds located in highly urban (at subplot level) areas were positively related with elevated pH values (Figure 4A). In addition, bacterioplankton communities in highly urbanized areas have a significantly greater variation among themselves compared to those in areas of medium and low urbanization (Figure 4B). When corrected for space and urbanization (at both scales), five variables were retained for the conditional environmental model (E|S+U) of the abundant fraction: pH, alkalinity, total phosphorus, abundance of Daphnia, and concentration of copper (Cu) (Table 3). For the dataset considering only the 12 most abundant OTUs, seven local environmental variables were selected for the uncorrected environmental model: pH, abundance of *Daphnia*, suspended matter, total nitrogen, maximum depth, molybdenum (Mo) and copper concentration (Cu) (Table 3). When corrected for urbanization at plot level, the conditional environmental model still retained all variables (Table 3).

**Table 3:** Results of RDA analyses.

Model	Significant environmental variables			
	E (marginal effect)	adj*R^2^*	E|S+U (conditional effect)	adj*R^2^*
Abundant (100 OTUs)	pH	0.035**	pH	0.027***
	Alkalinity	0.036***	Alkalinity	0.029***
	Total phosphorus	0.024**	Total phosphorus	0.016**
	Abundance of *Daphnia*	0.024**	Abundance of *Daphnia*	0.020**
	Conductivity	0.019**	Copper (Cu)	0.014*
	Copper (Cu)	0.013*		
Dominant (12 OTUs)	pH	0.065***	pH	0.029***
	Abundance of *Daphnia*	0.047**	Suspended matter	0.012*
	Suspended matter	0.044**	Total nitrogen	0.010*
	Molybdenum (Mo)	0.040**	Maximum depth	0.013*
	Copper (Cu)	0.028*	Abundance of *Daphnia*	0.016*
	Total nitrogen	0.016**	Copper (Cu)	0.010*
	Maximum depth	0.021*	Molybdenum (Mo)	0.013*


*Significant environmental variables identified by forward selection, explaining the variation (adj*R^2^*) in bacterioplankton community composition. Environmental variables effects were not (marginal effect) or were corrected (conditional effect) for the abundant (100 OTUs – relative abundance ≥ 0.1%) and in the dominant fraction (12 OTUs – relative abundance ≥ 1%). E, environment; E|S + U, environment corrected for space and urbanization (plot and subplot). ^∗∗∗^*P* < 0.001, ^∗∗^*P* < 0.01, ^∗^*P* < 0.05.*

Since pH was the variable explaining the highest variation in bacterioplankton communities (3.5% – abundant, 6.5% – dominant dataset), we further analyzed the effect of pH on bacterioplankton community composition along the studied urbanization gradient. Bacterioplankton community composition significantly varied along the pH gradient as confirmed by PERMANOVA (for categorical classification of pH < 7, 7–8, 8–9, and > 9, PERMANOVA, *P* < 0.01). Ponds with the highest pH levels, mostly in subplots characterized by a high level of urbanization, harbored clearly distinct bacterioplankton communities (Figure 1B). Further, we found a significant difference among pH values across urbanization subplots, with higher pH values in high urbanization subplots than in low urbanizations subplots (*P* = 0.024) (Figure 1C) (Supplementary Table S10). The other environmental variables selected by forward selection driving bacterioplankton community composition did not show significant differences across urbanization plots and subplots (Supplementary Figure S6).

## Discussion

We evaluated how and to what extent bacterioplankton communities of shallow ponds are affected by urbanization in a metacommunity context. Based on 50 shallow ponds sampled in Belgium, urbanization (measured as percentage of built-up area) turned out to have only a minor direct effect on bacterioplankton community composition. At local subplot level (200 m × 200 m), urbanization did not explain any significant portion of community variance, and at regional plot level (3 km × 3 km), urbanization explained only a small significant fraction of variation (1.9% for the abundant fraction, 3.4% for the dominant fraction). In contrast, the measured environmental variables explained 15.4–26.1% of the variation in bacterioplankton when corrected for confounding effects of urbanization. As we predicted, urbanization had an indirect effect on bacterioplankton community, apparently mediated by environmental factors. Also, at the level of OTU diversity, no significant effects of urbanization were observed, and relative abundances of the most common bacterioplankton classes did not differ among the urbanization categories, although significant differences were observed for two dominant OTUs.

These results are in contrast to the relatively strong effects of urbanization reported for birds, mammals and plants, which include biotic homogenization (McKinney, 2006) and reduced biodiversity (Grimm et al., 2008). The bacterioplankton communities in our study do not follow these trends, as we observed a significantly higher variation in bacterioplankton community composition among urban compared to rural and semi-natural sites at the subplot level (Figure 4B) and no effect on diversity on any of the spatial urbanization scales (Supplementary Figure S3). We see different potential reasons for this contrasting response to urbanization of bacterioplankton in shallow ponds. First, most ponds sampled in this study were man-made ponds and probably managed. Although we do not have management data of the sampled ponds, we know that management can influence pond variables such as nutrient state and macrophyte abundance (Declerck et al., 2006). Variation in pond management among ponds might have resulted in a similar gradient in pond types in both urban and rural areas, independent of urbanization category. We indeed observed a similar gradient in environmental conditions in both rural, semi-urban, and urban ponds (Supplementary Table S11). Second, ponds are inherently highly fragmented systems and can be seen as islands in a terrestrial matrix. Habitat fragmentation and reduced dispersal, associated with urbanization and impacting many terrestrial organisms (Alberti et al., 2017), might play a less important role in community assembly of bacterioplankton and small aquatic organisms than of larger organisms. It has recently been shown across studied areas in Manhattan (New York City, NY, United States) that microbial richness was not reduced by the effects of urbanization, while it had a pronounced effect on macro-organisms (Reese et al., 2015).

The small proportion of variation in community composition explained by urbanization at plot level was mostly shared with environment variables (1.9–3.4%). One environmental variable (pH) was correlated with the level of urbanization and did influence bacterioplankton community composition significantly (Figure 1C). pH is known to be an important driver for microbial communities related to species turnover in extreme conditions (Ren et al., 2015). It also plays a role in continuous gradients such as presented here and also observed in other studies (Mukherjee et al., 2014; Xu et al., 2014; Yan et al., 2016). In our dataset the pH ranged from 6.35 to 10.14, playing a significant role in structuring the bacterioplankton communities studied here. Urbanization is known to mediate pH values due to an increase of impervious surfaces in more urbanized areas (Conway, 2007). This increase could be related to the leaching of bicarbonates of concrete structures used in impervious surfaces in urban areas (Davies et al., 2010).

Also, *Daphnia* abundance contributed significantly to explain variation in bacterioplankton community composition. Experimental studies have previously found that the abundance of *Daphnia* can have an impact on bacterioplankton communities (Langenheder et al., 2001; Verreydt et al., 2012; Berga et al., 2015). Large-bodied water fleas of the genus *Daphnia* are pivotal species in the food web of ponds and lakes, as they can exert a strong top-down impact on microbial communities (Jürgens and Jeppesen, 2000; Degans and De Meester, 2002; Zöllner et al., 2003). Moreover, a symbiotic relationship between *Daphnia* species and some important genera of bacterioplankton has been reported (Zöllner et al., 2003; Qi et al., 2009; Callens et al., 2016). Some bacteria, such as of the genus *Limnohabitans* (one of the most abundant taxa found in our dataset), are an important component of freshwater bacterioplankton communities and have been found to be also part of the microbiota of *Daphnia* species (Callens et al., 2016). A parallel analysis of metacommunity structure of zooplankton in the same systems revealed a small but significant signature of urbanization on the abundance of Daphniidae (Gianuca et al., 2018). The selection of *Daphnia* abundance as a factor to explain variation in bacterioplankton community composition (even when corrected for urbanization) reinforces the importance to include biotic variables to better understand what drives bacterioplankton communities.

While other general pond characteristics such as conductivity, nutrients concentration, and abundance of *Daphnia* highly structured the bacterioplankton communities in the present dataset independently of urbanization, there are some small effects correlated to urbanization level. These effects on bacterioplankton community composition are less strong than we would have expected based on the studies in terrestrial and other aquatic organisms that were conducted within the same hierarchical design and field survey as the present study. A study on zooplankton using the same ponds also found that the effect of urbanization on community composition was significant but small (4%; Gianuca et al., 2018). In terrestrial carabid beetles, using slightly different subplots, changes in species composition were significantly related to urbanization at both spatial scales (Piano et al., 2017). Finally, a study analyzing community data from this same design across 10 macro-organisms found a strong effect of urbanization in shifts of body size (Merckx et al., 2018). It has recently been shown that the effects of urbanization on microbial organisms might be different compared to macro-organisms, where urbanization leads to reduced diversity and homogenization (Reese et al., 2015). For aquatic organisms such as micro-organisms that are not strongly dispersal limited, the ponds and lakes in urban areas can represent a broad range of environmental conditions due to the interplay of different abiotic and biotic factors and the release from interference from larger organisms which could allow more or different species to coexist. The level of exclusiveness for some taxa found in urban ponds further reinforces this notion (Figure 2A,B).

Our variation partitioning analyses further indicate that environmental rather than spatial factors accounted for the variation in bacterioplankton community structure. This suggests that species sorting was the main process explaining bacterioplankton community assembly, reinforcing previous observations that abiotic and biotic variables have a stronger effect on bacterioplankton community variation than regional factors across different spatial scales (Van der Gucht et al., 2007; Jones and McMahon, 2009; Logue et al., 2010; Souffreau et al., 2015). Urbanization seems to have an indirect effect mediating the relative importance of species sorting through water chemistry at subplot level. However, at plot level we detect a small, but direct effect on composition that could be related to an effect of urbanization on the bacterioplankton regional pool of species. These different effects of urbanization at subplot and plot level could suggest that urbanization act in distinct ways depending of the spatial scale. The relative importance of species sorting driving bacterioplankton communities has been related to the degree of environmental variation among sites (Östman et al., 2010), with higher environmental heterogeneity leading to an increase in the relative importance of species sorting. The selection of 6–7 different environmental variables in our environmental model show that along the studied urbanization gradient, multiple environmental variables were involved in explaining changes in community composition of bacterioplankton.

The relatively higher proportion of explained variation, when only considering the dominant taxa (relative abundances ≥ 1%) compared to the abundant taxa (relative abundances ≥ 0.1) is likely due to statistical constraints, since it is known that microbial communities have an elevated number of species compared to macro-organisms and consequently the inclusion of rare species, introduces noise to the patterns (Siqueira et al., 2012; Székely and Langenheder, 2014). It has also been shown before that the dominant patterns in the structure of microbial communities are indeed governed by a relatively small group of taxa (Székely and Langenheder, 2014). The selection of a similar set of environmental conditions determining bacterioplankton structure for both fractions studied (Table 3) reinforces this idea.

Based on our results, bacterioplankton communities in shallow ponds along an urbanization gradient were structured by processes (i.e., species sorting) predominantly acting at the level of the individual pond. This result is similar to the one previously found with respect to agricultural land use intensity structuring different aquatic organisms in the same sampling area (De Bie et al., 2012). Environmental conditions may be more important than neutral processes for these aquatic microorganisms. pH was shown to be an important environmental factor and was mediated by urbanization. The greater dispersal capacity of microbes compared to macro-organisms could make them less susceptible to impacts of landscape fragmentation due to urbanization. Instead, changes in local conditions mediated by urbanization have a greater impact on bacterioplankton community composition. Species sorting is more likely the most important process explaining community variation of bacterioplankton along urbanization gradients. Analysis of the functional gene composition of bacterioplankton communities through metagenomics would give us more in-depth information on the impact of urbanization on bacteria beyond taxonomy.

## Author Contributions

FH, CS, JE, KB, and LDM designed the research. FH, CS, JE, and KB performed the research. FH, SM, CS, and PB analyzed the data. FH, SM, CS, and LDM wrote the manuscript. All the authors edited and approved the manuscript.

## Conflict of Interest Statement

The authors declare that the research was conducted in the absence of any commercial or financial relationships that could be construed as a potential conflict of interest.

## Footnotes

1 http://www.mothur.org/wiki/Schloss_SOP
