# Supplementary material for: Freshwater Bacterioplankton Metacommunity Structure Along Urbanization Gradients in Belgium
Source: Front Microbiol. 2019 Apr 12;10:743. doi: 10.3389/fmicb.2019.00743 (PMC6473040; doi:10.3389/fmicb.2019.00743)
Supplement: Supplementary file 1 [file Table_1.docx]

Supplementary Material

Freshwater bacterioplankton metacommunity structure along urbanization gradients in Belgium

Fabio Toshiro. T. Hanashiro*, Shinjini Mukherjee, Caroline Souffreau, Jessie Engelen, Kristien I. Brans, Pieter Busschaert, Luc De Meester

*** Correspondence:** Fabio Toshiro. T. Hanashiro: fabiotoshiro@gmail.com

## Content

Supplemental Figure S1

Supplemental Table S1

Supplemental Table S2

Supplemental Figure S2

Supplemental Table S3

Supplemental Table S4

Supplemental Table S5

Supplemental Table S6

Supplemental Table S7

Supplemental Figure S3

Supplemental Table S8

Supplemental Figure S4

Supplemental Figure S5

Supplemental Table S9

Supplemental Table S10

Supplemental Figure S6

Supplemental Table S11

**
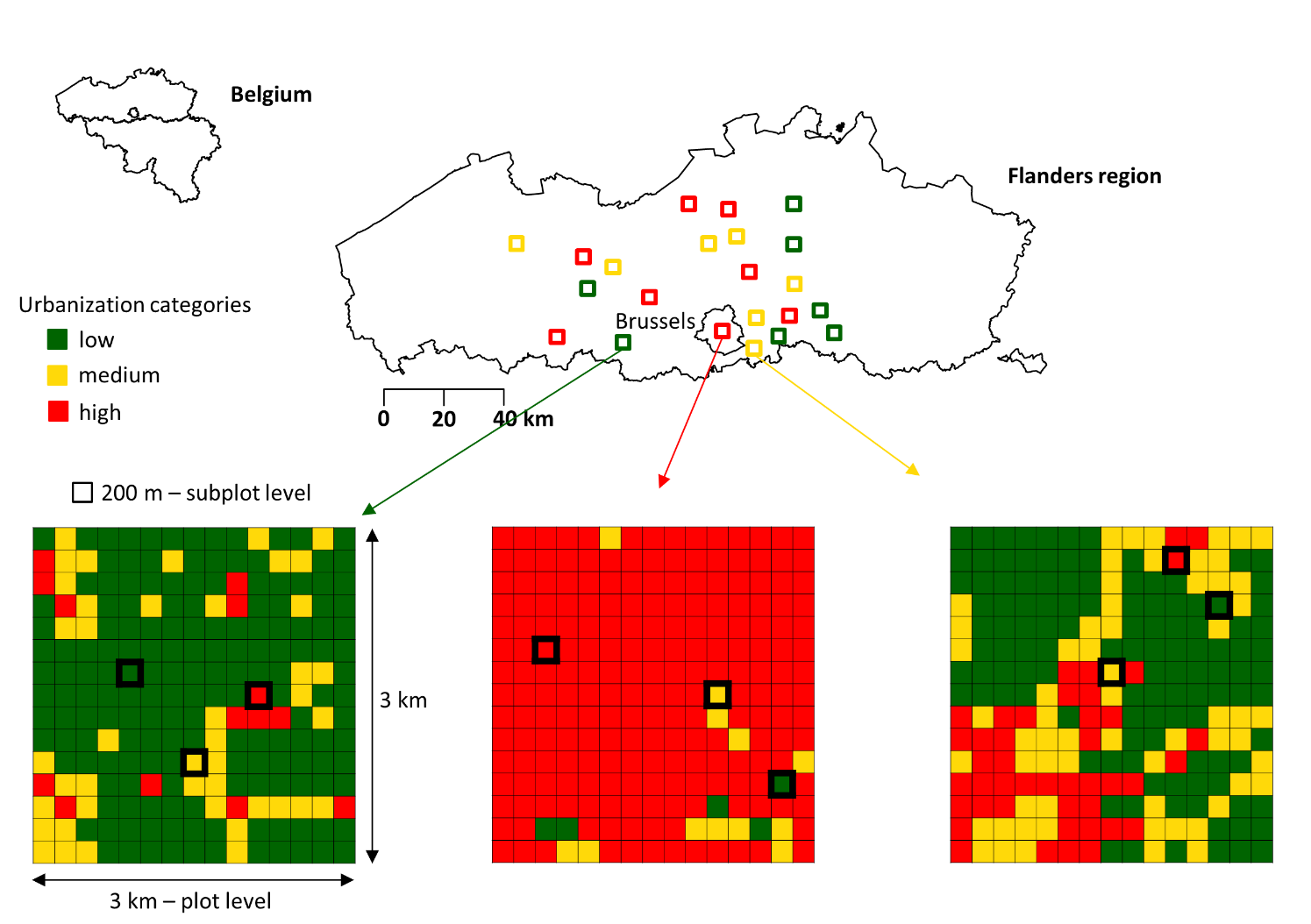
**

**Figure S1.** Localization of the Flanders region in Belgium and the representation of the hierarchical design; large squares represent 3 x 3 km plots, with grids of 200 m x 200 m subplots within plots, locations of ponds indicated as bold squares. Colors reflect urbanization categories: low (green), medium (yellow) and high (red). A detailed description of the hierarchical design is described in the main text. Maps of Belgium and Flanders region were produced in R using the package *BelgiumMaps.StatBel*.

**Table S1.** Summary of the number of plots, subplot and combination of both levels of urbanization for the 50 sites sampled.

|  | Urbanization levels |  |  |
| --- | --- | --- | --- |
|  | Low | Medium | High |
| Plot | 17 | 13 | 20 |
| Subplot | 15 | 18 | 17 |
| Combination | Low (Plot) | Medium (Plot) | High (Plot) |
| Low (Subplot) | 3 | 7 | 7 |
| Medium (Subplot) | 6 | 3 | 4 |
| High (Subplot) | 6 | 8 | 6 |

**Table S2.** Morphometric data for the 50 sites sampled.

|  | Morphometric variables | |  |  |
| --- | --- | --- | --- | --- |
| Pond codes | Depth (m) / Transparency (m) | Surface area (m^2^) | Average bank angle (cm) | Average depth at edge (cm) |
| PL1-YEL  PL10-GRE  PL10-RED  PL10-YEL  PL11-GRE  PL11-RED  PL11-YEL  PL12-GRE  PL12-RED  PL13-GRE  PL13-RED  PL13-YEL  PL14-RED  PL14-YEL  PL15-GRE  PL15-RED  PL15-YEL  PL16-GRE  PL16-RED  PL18-YEL  PL2-GRE  PL2-RED  PL2-YEL  PL23-GRE  PL23-RED  PL23-YEL  PL24-RED  PL24-YEL  PL25-RED  PL25-YEL  PL26-GRE  PL26-RED  PL26-YEL  PL27-GRE  PL27-RED  PL27-YEL  PL3-GRE  PL3-RED  PL3-YEL  PL4-GRE  PL4-RED  PL5-YEL  PL6-RED  PL6-YEL  PL7-GRE  PL7-RED  PL7-YEL  PL8-GRE  PL9-GRE  PL9-YEL | 1.5 / 0.5  0.75 / 0.25  1.3 / 0.30  1.2 / 0.16  1.42 / 0.43  1.1 / 0.35  1.4 / 0.29  0.7 / 0.17  2 / 0.22  1.8 / 0.18  0.7 / 0.05  0.6 / 0.5  1.2 / 0.05  2 / 0.22  1 / 0.14  1 / 0.27  1.8 / 0.21  0.6 / 0.30  0.3 / 0.21  0.65 / 0.24  1.5 / 0.22  1.5 / 0.19  1.5 / 0.43  1 / 0.12  0.75 / 0.29  2 / 0.18  1 / 0.26  1.5 / 0.22  0.7 / 0.11  0.7 / 0.28  0.85 / 0.22  1.75 / 0.20  1 / 0.17  0.7 / 0.11  1 / 0.13  1 / 0.13  0.8 / 0.23  1 / 0.25  0.5 / 0.10  1.2 / 0.15  0.8 / 0.23  1.5 / 0.14  1.5 / 0.40  2.5 / 0.23  0.44 / 0.09  1.8 / 0.43  1.5 / 0.07  1.7 / 0.16  2 / 0.15  3 / 0.41 | 1306  12  31  589  294  1225  628  75  1200  1922  117  314  54  109  365  1963  292  87  176  471  82  1100  375  58  56  6400  151  31  56  95  39  3675  628  282  0.047  62  37  157  43  1750  27  300  157  942  400  144  37  484  1280  240 | 70  25  40  90  15  65  45  40  35  10  5  30  55  60  10  40  80  40  90  35  35  45  80  75  90  30  50  50  60  40  20  35  15  50  5  45  15  15  80  30  45  25  90  90  5  80  50  30  28  60 | 60  5  15  40  10  15  15  10  10  10  10  10  25  15  10  10  50  8.5  5  7  30  20  20  12  70  10  20  25  30  14  8  20  13  7  0  10  15  15  15  10  12  10  20  30  8  40  10  15  10  40 |

**Figure S2.** Rarefaction curves for each sample. Rarefaction curves are based on the normalized datasets (688 seq/sample) with OTUs>0.1% of the total relative abundance (100 OTUs).

**Table S3.** Summary of the RDA analyses showing the relative importance of the different components of explained variation of community composition in the abundant (100 OTUs - relative abundance ≥0.1%) and in the dominant fraction (12 OTUs - relative abundance ≥1%) for the coverage of 1000 sequences per sample (38 samples). The effects of each component (E – environment, S – space, U – urbanization at subplot and plot levels) are not (marginal effect) or are corrected (conditional effect) for the other components. NS means that a certain model did not explain the variation in community composition significantly, or that the % explained variance was not significant.

| Model | Components studied | | | | | | | |
| --- | --- | --- | --- | --- | --- | --- | --- | --- |
|  | Marginal effect | | | | Conditional effect | | | |
|  | E | S | U(subplot) | U(plot) | E | S | U(subplot) | U(plot) |
| Abundant (107 OTUs) | 0.184*** | NS | NS | 0.018* | 0.167 *** | NS | NS | 0.013^NS^ |
| Dominant (12 OTUs) | 0.302*** | NS | NS | 0.057* | 0.277*** | NS | NS | 0.032* |

E – Environment, S – Space, U – Urbanization (NS – not significant, ****p* < 0.001, **p* < 0.05)

**Table S4.** Results of RDA analyses. Significant environmental variables identified by forward selection, explaining the variation (adj*R^2^*) in bacterioplankton community composition. Environmental variables effects were not (marginal effect) or were corrected (conditional effect) for the abundant (100 OTUs - relative abundance ≥0.1%) and in the dominant fraction (12 OTUs - relative abundance ≥1%) for the coverage of 1000 sequences per sample (38 samples).

| Model | Significant environmental variables | | |  |
| --- | --- | --- | --- | --- |
|  | E (marginal effect) | adj*R^2^* | E\|S+U  (conditional effect) | adj*R^2^* |
| Abundant  (107 OTUs) | pH  alkalinity  conductivity  total phosphorus  dissolved organic carbon  abundance of *Daphnia* | 0.041***  0.036**  0.034**  0.034**  0.019*  0.020* | pH  alkalinity  dissolved organic carbon  total phosphorus  abundance of *Daphnia* | 0.036***  0.038***  0.018*  0.019*  0.017* |
| Dominant  (12 OTUs) | pH  abundance of *Daphnia*  suspended matter  total nitrogen  molybdenum (Mo)  maximum depth  nickel (Ni) | 0.078***  0.051**  0.050**  0.032**  0.031*  0.035*  0.025* | pH  suspended matter  total nitrogen  maximum depth  abundance of *Daphnia*  molybdenum (Mo) | 0.039***  0.019**  0.016*  0.015*  0.017**  0.013* |

E – Environment, E|S + U – Environment corrected for space and urbanization (plot and subplot)

(****p* < 0.001, ***p* < 0.01, **p* < 0.05)

**Table S5.** Results of the test for differences in community composition in the abundant fraction (100 OTUs - relative abundance ≥0.1%) for the coverage of 1000 sequences per sample (38 samples) at the plot and subplot levels as inferred from PERMANOVA analysis.

|  | SS | MS | F | R^2^ | *P* |
| --- | --- | --- | --- | --- | --- |
| U_plot_ | 0.56 | 0.28 | 1.54 | 0.07 | 0.02 |
| U_subplot_ | 0.42 | 0.21 | 1.15 | 0.05 | 0.24 |
| U_plot_: U_subplot_ | 0.87 | 0.21 | 1.20 | 0.12 | 0.16 |
| Residuals | 5.27 | 0.18 |  | 0.73 |  |

**Table S6.** Results of the test for differences in community composition in the abundant fraction (100 OTUs - relative abundance ≥0.1%) at the plot and subplot levels as inferred from PERMANOVA analysis.

|  | SS | MS | F | R^2^ | *P* |
| --- | --- | --- | --- | --- | --- |
| U_plot_ | 0.59 | 0.29 | 1.62 | 0.06 | 0.01 |
| U_subplot_ | 0.41 | 0.20 | 1.11 | 0.04 | 0.27 |
| U_plot_: U_subplot_ | 0.79 | 0.19 | 1.07 | 0.08 | 0.29 |
| Residuals | 7.52 | 0.18 |  | 0.80 |  |

**Table S7.** Results of the test for the response of bacterioplankton diversity in relation to urbanization at plot (U_plot_) and subplot level (U_subplot_).

| Fixed effects | U_plot_ |  | U_subplot_ |  | U_plot_: U_subplot_ |  |
| --- | --- | --- | --- | --- | --- | --- |
| Factor | F | P | F | P | F | P |
| Richness | F_2,19_=0.79 | 0.46 | F_2,22_=0.55 | 0.58 | F_4,22_=0.14 | 0.96 |
| Shannon Diversity | F_2,19_=0.72 | 0.49 | F_2,22_=0.06 | 0.93 | F_4,22_=0.98 | 0.43 |
| Chao1 | F_2,19_=0.09 | 0.91 | F_2,22_=0.13 | 0.87 | F_4,22_=0.40 | 0.80 |
| ACE | F_2,19_=0.38 | 0.68 | F_2,22_=0.33 | 0.71 | F_4,22_=0.48 | 0.75 |

**Figure S3.** Box-plots of different diversity indices (Richness, Shannon, ACE and Chao1) along urbanization at subplot and plot level (x-axis). Urbanization categories at subplot level are represented by colors within the three categories at plot level (low, medium and high) depicted in the x-axis.

**Table S8.** Results of the test for the response for the ten most abundant classes found in relation to urbanization at plot (U_plot_) and subplot level (U_subplot_).

| Fixed effects | U_plot_ |  | U_subplot_ |  | U_plot_: U_subplot_ |  |
| --- | --- | --- | --- | --- | --- | --- |
| Factor | F | P | F | P | F | P |
| Betaproteobacteria | F_2,19_=0.76 | 0.47 | F_2,22_=0.0003 | 1.00 | F_4,22_=0.45 | 0.76 |
| Actinobacteria | F_2,19_=0.67 | 0.52 | F_2,22_=0.30 | 0.73 | F_4,22_=1.29 | 0.30 |
| Bacteroidetes | F_2,19_=0.39 | 0.67 | F_2,22_=0.10 | 0.89 | F_4,22_=1.53 | 0.22 |
| Alphaproteobacteria | F_2,19_=3.27 | 0.059 | F_2,22_=0.95 | 0.40 | F_4,22_=0.43 | 0.78 |
| Unclassified | F_2,19_=0.63 | 0.53 | F_2,22_=3.27 | 0.056 | F_4,22_=4.07 | 0.01 |
| Gammaproteobacteria | F_2,19_=0.82 | 0.45 | F_2,22_=1.64 | 0.21 | F_4,22_=1.52 | 0.22 |
| Firmicutes | F_2,19_=1.54 | 0.23 | F_2,22_=1.20 | 0.31 | F_4,22_=2.72 | 0.55 |
| Epsilonproteobacteria | F_2,19_=0.65 | 0.53 | F_2,22_=0.43 | 0.65 | F_4,22_=1.09 | 0.38 |
| Unclassified (Proteo) | F_2,19_=0.70 | 0.50 | F_2,22_=2.54 | 0.10 | F_4,22_=0.60 | 0.66 |
| Deltaproteobacteria | F_2,19_=0.85 | 0.44 | F_2,22_=0.05 | 0.94 | F_4,22_=1.32 | 0.29 |

**Figure S4.** Box-plots of six abundant classes found along the urbanization gradient at subplot and plot levels. Urbanization categories at subplot level are represented by colors within the three categories at plot level (low, medium and high) depicted in the x-axis.

**Figure S5.** Box-plots of the 12 dominant OTUs (> 1% of local abundance) found along the urbanization gradient at subplot and plot levels. Urbanization categories at subplot level are represented by colors within the three categories at plot level (low, medium and high) depicted in the x-axis.

**Table S9.** Results of the test for the response of 12 dominant OTUs (> 1% of local abundance) in relation to urbanization at plot (U_plot_) and subplot level (U_subplot_).

| Fixed effects | U_plot_ |  | U_subplot_ |  | U_plot_: U_subplot_ |  |
| --- | --- | --- | --- | --- | --- | --- |
| Factor | F | P | F | P | F | P |
| Agrococcus | F_2,19_=1.09 | 0.35 | F_2,22_=0.79 | 0.46 | F_4,22_=0.53 | 0.71 |
| unclassified_1 | F_2,19_=0.80 | 0.46 | F_2,22_=1.08 | 0.35 | F_4,22_=2.68 | 0.05 |
| Limnohabitans_1 | F_2,19_=0.13 | 0.87 | F_2,22_=0.36 | 0.69 | F_4,22_=0.59 | 0.66 |
| Polynuclobacter_1 | F_2,19_=0.56 | 0.57 | F_2,22_=1.49 | 0.24 | F_4,22_=0.34 | 0.84 |
| Limnohabitans_2 | F_2,19_=0.39 | 0.68 | F_2,22_=1.63 | 0.21 | F_4,22_=0.64 | 0.63 |
| Cytophagaceae | F_2,19_=0.46 | 0.63 | F_2,22_=0.63 | 0.54 | F_4,22_=0.46 | 0.76 |
| unclassified (Actinobacteria-class) | F_2,19_=7.09 | 0.005 | F_2,22_=3.96 | 0.03 | F_4,22_=6.83 | 0.001 |
| Polynuclobacter_2 | F_2,19_=0.48 | 0.62 | F_2,22_=2.27 | 0.12 | F_4,22_=1.24 | 0.32 |
| Methylobacter | F_2,19_=0.40 | 0.67 | F_2,22_=8.84 | 0.001 | F_4,22_=8.05 | 0.004 |
| Acinetobacter | F_2,19_=1.14 | 0.34 | F_2,22_=1.50 | 0.24 | F_4,22_=0.64 | 0.63 |
| unclassified_2 | F_2,19_=0.84 | 0.44 | F_2,22_=2.38 | 0.11 | F_4,22_=3.62 | 0.02 |
| Flavobacterium | F_2,19_=0.68 | 0.51 | F_2,22_=0.45 | 0.63 | F_4,22_=0.45 | 0.77 |

**Table S10.** Results of the test for the response of the six environmental factors selected by forward selection driving bacterioplankton community composition in relation to urbanization at plot (U_plot_) and subplot level (U_subplot_).

| Fixed effects | U_plot_ |  | U_subplot_ |  | U_plot_: U_subplot_ |  |
| --- | --- | --- | --- | --- | --- | --- |
| Factor | F | P | F | P | F | P |
| pH | F_2,19_=0.75 | 0.48 | F_2,22_=4.4 | 0.02 | F_4,22_=2.62 | 0.06 |
| Alkalinity | F_2,19_=3.15 | 0.06 | F_2,22_=0.78 | 0.46 | F_4,22_=0.56 | 0.69 |
| Conductivity | F_2,19_=2.33 | 0.12 | F_2,22_=1.11 | 0.34 | F_4,22_=0.65 | 0.63 |
| Abundance of *Daphnia* | F_2,19_=0.36 | 0.69 | F_2,22_=1.98 | 0.16 | F_4,22_=1.18 | 0.34 |
| Total phosphorus | F_2,19_=1.05 | 0.36 | F_2,22_=2.17 | 0.13 | F_4,22_=0.08 | 0.98 |
| Copper concentration | F_2,19_=0.47 | 0.62 | F_2,22_=2.10 | 0.14 | F_4,22_=0.11 | 0.97 |

**Figure S6.** Box-plots showing the variation of the six environmental factors selected by forward selection driving bacterioplankton community composition among the urbanization categories at subplot and plot level. Urbanization categories at subplot level are represented by colors within the three categories at plot level (low, medium and high) depicted in the x-axis.

**Table S11**. Values of the environmental variables explaining variation in bacterioplankton community composition selected by forward selection for the 50 sites sampled.

|  | Selected variables | |  |  |  |  |
| --- | --- | --- | --- | --- | --- | --- |
| Pond codes | Conductivity (µS) | pH | Alkalinity (mg/L) | Total phosphorus (mg/L) | Abundance of *Daphnia* (ind/L) | Copper concentration |
| PL1-YEL  PL10-GRE  PL10-RED  PL10-YEL  PL11-GRE  PL11-RED  PL11-YEL  PL12-GRE  PL12-RED  PL13-GRE  PL13-RED  PL13-YEL  PL14-RED  PL14-YEL  PL15-GRE  PL15-RED  PL15-YEL  PL16-GRE  PL16-RED  PL18-YEL  PL2-GRE  PL2-RED  PL2-YEL  PL23-GRE  PL23-RED  PL23-YEL  PL24-RED  PL24-YEL  PL25-RED  PL25-YEL  PL26-GRE  PL26-RED  PL26-YEL  PL27-GRE  PL27-RED  PL27-YEL  PL3-GRE  PL3-RED  PL3-YEL  PL4-GRE  PL4-RED  PL5-YEL  PL6-RED  PL6-YEL  PL7-GRE  PL7-RED  PL7-YEL  PL8-GRE  PL9-GRE  PL9-YEL | 117.5  546  1166  956  520  522  788  387  369  724  11787  388  103.8  1152  568  1055  1072  167.2  270  862  164.3  659  492  29.7  308  538  290  214.6  692  368  409  127.2  164.8  309  220  259  860  463  479  275  278  439  751  242  108.2  112  133  489  1277  677 | 8.22  8.2  7.85  7.96  7.85  7.84  8.04  6.89  10.14  7.83  7.1  8.38  9.88  7.64  8.64  8.19  7.92  7.63  7  7.81  6.7  7.27  7.48  6.55  8.09  7.67  8.91  7.65  8  8.24  7.9  7.16  7.55  7.53  8.46  7.56  7.37  7.47  8.02  6.83  7.6  7.845  7.7  7.96  6.62  9.39  6.35  7.65  7.77  7.73 | 170  285  225  270  245  245  390  155  55  330  200  185  115  550  230  225  385  75  175  370  60  160  200  75  170  180  140  175  275  195  180  265  115  180  160  145  245  200  145  70  155  145  305  40  75  45  60  150  385  385 | 0.04559771  0.41561286  0.09781234  0.3346269  0.2519048  0.16830728  0.69505283  0.34007148  0.04406831  1.02733871  0.82602529  0.03955927  0.74173638  1.88222001  0.08615264  0.27429968  2.21088384  0.18261595  0.04509036  2.10057272  0.17170041  0.12772228  0.08245156  0.3548875  0.04785223  0.35106684  0.05251156  0.75556042  0.34752642  0.0355312  0.58058345  0.07635301  1.93581623  0.87018378  0.2369348  1.12912268  0.14274939  1.86279623  1.62861323  0.23443612  0.16613926  4.23545085  0.03047741  0.05386427  0.67373645  0.55908369  1.24815754  0.26064865  0.20112938  0.07078819 | 0  0  40.3125  55.04  19.17083333  6.45  114.1888889  0  0  75.035  3.225  0  0  3.225  0.04673913  49.62916667  193.2133333  157.38  0  272.3333333  5.52857143  0  59.81777778  27.48928571  0.05  0  0  0  113.5916667  0.775  0  0  345.075  0  0  7.525  0.125  1.775  405.6333333  0  0.1  19.03382353  31.7125  0  376.25  0.125  104.8125  253.7  0  0.35833333 | 0.01  0.09  0.06  0.08  0.09  0.11  0.05  0.01  0.33  0.03  0.02  0.04  0.02  0  0.16  0.17  0.06  0.12  0.04  0.1  0.07  0.07  0.07  0.02  0.02  0.02  0.33  0.04  0.05  0.11  0.08  0.09  0.16  0.28  0.75  0.06  0.18  0.03  0.05  0.06  0.03  0.03  0.02  0.05  0.2  0.61  0.07  0.05  0.05  0.05 |
